# A secretome profile indicative of oleate-induced proliferation of HepG2 hepatocellular carcinoma cells

**DOI:** 10.1038/s12276-018-0120-3

**Published:** 2018-08-03

**Authors:** Soyeon Park, Ji-Hwan Park, Hee-Jung Jung, Jin-Hyeok Jang, Sanghyun Ahn, Younah Kim, Pann-Ghill Suh, Sehyun Chae, Jong Hyuk Yoon, Sung Ho Ryu, Daehee Hwang

**Affiliations:** 1 Department of Life Sciences, Pohang University of Science and Technology (POSTECH), Pohang, Kyungbuk 37673 Republic of Korea; 2 Center for Plant Aging Research, Institute for Basic Science (IBS), Daegu, 42988 Republic of Korea; 3 Department of Brain and Cognitive Sciences, Gyeongbuk Institute of Science and Technology (DGIST), Daegu, 42988 Republic of Korea; 4 School of Life Sciences, Ulsan National Institute of Science and Technology, Ulsan, 44919 Republic of Korea; 5 Department of Neural Development and Disease, Korea Brain Research Institute, Daegu, 41068 Republic of Korea; 6 Department of New Biology, DGIST, Daegu, 42988 Republic of Korea

## Abstract

Increased fatty acid (FA) is often observed in highly proliferative tumors. FAs have been shown to modulate the secretion of proteins from tumor cells, contributing to tumor survival. However, the secreted factors affected by FA have not been systematically explored. Here, we found that treatment of oleate, a monounsaturated omega-9 FA, promoted the proliferation of HepG2 cells. To examine the secreted factors associated with oleate-induced cell proliferation, we performed a comprehensive secretome profiling of oleate-treated and untreated HepG2 cells. A comparison of the secretomes identified 349 differentially secreted proteins (DSPs; 145 upregulated and 192 downregulated) in oleate-treated samples, compared to untreated samples. The functional enrichment and network analyses of the DSPs revealed that the 145 upregulated secreted proteins by oleate treatment were mainly associated with cell proliferation-related processes, such as lipid metabolism, inflammatory response, and ER stress. Based on the network models of the DSPs, we selected six DSPs (MIF, THBS1, PDIA3, APOA1, FASN, and EEF2) that can represent such processes related to cell proliferation. Thus, our results provided a secretome profile indicative of an oleate-induced proliferation of HepG2 cells.

## Cancer: secreted proteins associated with fat-induced tumor growth

By exposing liver cancer cells to oleate, an unsaturated fatty acid, researchers have discovered a group of secreted proteins that may help explain why fatty acids increase proliferative capacity in tumors. Soyeon Park from Pohang University of Science and Technology in South Korea and coworkers treated liver cancer cells with oleate and then measured all the proteins released from the cells. Comparison with untreated cells revealed 145 proteins secreted at elevated levels—most of which were involved in metabolism, stress responses and other proliferation-related processes—and another 192 proteins secreted at reduced levels. The researchers ran additional biochemical analyses on six secreted proteins to validate the changes following exposure to oleate. The authors suggest that these validated proteins could now serve as biomarkers of tumor aggressiveness or as future drug targets.

## Introduction

Various factors are secreted from tumor cells, as well as other types of cells interacting with tumor cells, contributing to promoting or inhibiting tumor growth and survival. A number of proteomic analyses of secretomes have been performed for pancreatic, breast, prostate, bladder, and liver cancers^1–5^ to catalog the factors secreted from tumor cells. These analyses have mainly focused on the identification of proteins differentially secreted between tumor and normal cells and then proposed these proteins as potential diagnostic biomarkers for the cancers analyzed. However, tumor secretomes vary with different pathophysiological conditions, thereby altering tumor growth, survival, and/or invasion. A comparative proteomic analysis of tumor secretomes between different pathophysiological conditions has rarely been performed to understand alterations in the secreted factors associated with cancer pathogenesis.

Fatty acids (FAs) have been reported to affect the secretomes from tumors^6–8^. For example, linoleic acid enhanced the secretion of the plasminogen activator inhibitor-1 in breast cancer^6^, and oleate, a monounsaturated omega-9 FA, induced the secretion of matrix metallopeptidase 9 in breast cancer cells to promote their invasiveness^7^. Additionally, palmitate increased the secretion of interleukin-8 in steatotic hepatoma cells^8^, providing a higher potential for hepatic inflammation. Among the FAs, oleate was reported to be the most abundant circulating free FA in mammals^9–13^, and its level is often increased in cancer tissues^14^. The effect of oleate on the proliferation of cancer cells has been controversial. Many studies showed that oleate promoted the proliferation of cancer cells in various types of cancers^15,16^, but other studies showed the opposite effect. These contradictory observations are probably due to the differences in types of cancer cells, degree of malignancy, growth conditions, and/or even assay methods. Nevertheless, it has been consistently observed that oleate has substantial effects on the growth and survival of cancer cells. As aforementioned, oleate modulates the secretion of proteins from tumor cells, including cytokines and chemokines, which can contribute to the proliferation of cancer cells.

Accordingly, the investigation of secretory factors modulated by oleate is important to understand the effect of oleate on cell proliferation. However, these secretory factors affected by oleate still remain elusive. Here, to examine secretory factors affected by oleate, we performed a comparative secretome analysis of hepatocarcinoma HepG2 cells by profiling the proteomes of conditioned media collected with and without oleate treatment, using label-free liquid chromatography-tandem mass spectrometry (LC-MS/MS) analysis. HepG2 cells were used because they have been shown to secrete a broad spectrum of molecules (e.g., proteins and metabolites)^17–19^ and are widely used for various studies, including mechanism studies, drug screening, and secretome analysis^15,20–22^. The comparative secretome analysis of oleate-treated and untreated HepG2 cells identified 349 differentially secreted proteins (DSPs) by oleate treatment that are associated with cellular processes related to cell proliferation. Thus, our proteome data provide a secretome profile that can represent the cellular processes related to oleate-induced proliferation of HepG2 cells.

## Materials and methods

### Reagents and cell culture

Sodium oleate (O7501, St. Louis, MO) and sodium palmitate (P9767, St. Louis, MO) were purchased from Sigma-Aldrich. Bovine serum albumin, fraction V, and fatty acid-free (126575, San Diego, CA) was purchased from Calbiochem. Oleate or palmitate was dissolved in 100% methanol. After conjugation with fatty-acid-free BSA at a 5:1 fatty acid to BSA molar ratio, as previously described^23^, it was diluted to a proper final concentration in serum-free Minimum Essential Medium (MEM) just before the treatment of cells. HepG2 cells were grown in MEM (Welgene, LM 007-07) and then supplemented with 10% (v/v) fetal bovine serum (Lonza), 2 mM glutamine, and 1% penicillin-streptomycin (Gibco) at 37 °C, 5% CO_2_, and 95% humidity.

### Cell proliferation assays

Cell proliferation was measured by a colorimetric assay for cell viability using MTT (Sigma Aldrich, M5655). HepG2 cells were seeded in a 96-well culture plate and incubated for 24 h. After starvation with unsupplemented media for 12 h, cells were incubated with fatty acids treatment for 24 h, as done for the sample preparation of the secretome analysis. For control samples, cells were incubated with the equivalent concentrations of methanol and BSA contained in the fatty acid treatment conditions. Cells were treated with 0.5 mg/ml MTT reagent that was diluted in phenol-red-free media and incubated in a 37 °C CO_2_ incubator. After 2 h, the solution was replaced with 100 μl of DMSO to solubilize formazan crystals. The intensity was measured in plates at 540 nm absorbance by using a microplate reader.

### Sample preparation

HepG2 cells were starved for 12 h before fatty acid treatment. To eliminate secretory factors from cells during starvation, cells were washed three times with 1× PBS. For oleate-treated samples, BSA-conjugated oleate diluted to a final concentration of 500 μM in serum-free media was treated for 24 h. For control samples, oleate-free serum-free medium containing equivalent amounts of BSA and methanol used for oleate-treated samples was treated for 24 h. The conditioned media were prepared by replacing the media with unsupplemented serum-free media and incubating for 24 h in a 37 °C CO_2_ incubator. After centrifuging the conditioned media at 3000 r.p.m. for 10 min at 4 °C, only supernatants were collected and filtered using centrifugal filter units (Millipore, UFC900324) to remove contaminants. Samples were dried by utilizing a lyophilizer.

### Protein extraction and digestion

The samples were dried and then homogenized with lysis buffer (urea, NaCl, 50 mM Tris-HCl (pH 8.2) and a Complete Mini protease inhibitor tablet (Roche Applied Science, Basel, Switzerland)). The lysate was ultracentrifuged at 45,000×*g* at 4 °C for an hour. The protein concentration was determined from the resulting supernatant by the DC protein assay (Bio-Rad, Hercules, USA). The protein sample was then reduced in 0.5 M DTT (100 µl) for 50 min at 37 °C, followed by addition of 1.5 µl of 1 M iodoacetamide and incubation in the dark for 30 min at room temperature for alkylation. The resulting sample was subjected to in-solution tryptic digestion (1:50 enzyme-to-protein ratio (w/w), Promega, Madison, WI, USA) and then incubated at 37 °C overnight. The peptide samples were centrifuged at 2500×*g* for 10 min at room temperature. The resulting aqueous solution was desalted using solid-phase extraction with reverse-phase tC18 Sep-Pak solid-phase extraction cartridges as previously described^24^. Finally, the peptides were eluted with 50% ACN and 0.5% HAcO and then dried in a Speed-Vac with resuspension in 0.1% formic acid. The samples were stored at −20 °C before LC-MS/MS analysis.

### LC-MS/MS analysis

All peptide samples were separated on a Thermo EASY-nLC 1000 (Thermo Scientific, Odense, Denmark)^25^ equipped with analytical columns (Thermo Scientific, Easy-Column, 75 μm × 50 cm) and trap columns (75 μm × 2 cm). The operation temperature of the analytical columns was 50 °C. The flow rate was set to 300 nl/min. Solvent A was 0.1% formic acid and 2% acetonitrile in water, and solvent B was 0.1% formic acid and 2% water in acetonitrile. For the global proteome analysis, a 120-min gradient (from 2 to 40% solvent B over 90 min, from 40 to 80% solvent B over 10 min, 80% solvent B for 10 min and from 80 to 2% solvent B over 10 min) was used. The eluted peptides from LC were analyzed using the Q-Exactive™ hybrid quadrupole-Orbitrap mass spectrometer (Thermo Scientific)^26^ equipped with the nanoelectrospray source. For ionization of the eluting peptides, the electric potential of the electrospray ionization was kept at 1.7 kV, and the temperature of the desolvation capillary was set to 270 °C. The Q-Exactive mass spectrometer was operated in the data-dependent mode, with survey scans acquired for the mass range of 450–2000 Thomsons (Th) at a resolution of 70,000 (at *m*/*z* 200). The top 10 most abundant ions from the survey scan were selected for MS/MS analysis with an isolation window of 2.0 Th and fragmented by the higher energy collisional dissociation (HCD)^27^ with the normalized collision energies of 25 and the exclusion duration of 10 s. The MS/MS scans were acquired at a resolution of 17,500. Maximum ion injection times were 100 ms and 50 ms for the full MS and the MS/MS scan, respectively. The automated gain control (AGC) target value was set to 1.0 × 10^6^ and 1.0 × 10^5^ for the full MS and MS/MS scan, respectively.

### Peptide identification

The fragmentation spectra were created based on the mzXML file using the MSConvert tool (ProteoWizard release: 3.0.4323). MS data were first analyzed using postexperiment-monoisotopic mass refinement (PE-MMR) to assign an accurate precursor mass to tandem MS data^28^. MS/MS spectra were searched against a composite database containing UniProtKB/Swiss-Prot entries of the human reference proteome (UniProtKB release 2015_11, 42,123 entries) and 179 common contaminants in the target-decoy setting using the MS-GF + Beta (v10089) search engine^29^ under the following parameters: semitryptic, precursor mass tolerance of 10 p.p.m., carbamidomethylation of cysteine as a fixed modification and oxidation of methionine as a variable modification. The search results of 24 LC-MS/MS datasets were all combined, and the target-decoy analysis was performed on the combined dataset to obtain peptides with the false discovery rate (FDR) ≤ 0.01.

### Label-free peptide quantification

To assign an MS intensity to peptide identification, an MS intensity-based label-free quantification method was applied to 24 LC-MS/MS datasets as described previously^30^. Briefly, through PE-MMR analysis, MS features of a peptide that appeared over a period of LC elution time in an LC-MS/MS experiment were grouped into a unique mass class (UMC)^28^. Ideally, each UMC corresponds to a peptide and contains the ions corresponding to the peptide, together with their mass spectral features, such as charge states, abundances (intensity), scan numbers, and measured monoisotopic masses. For each UMC, we obtained the UMC mass by calculating the intensity-weighted average of the monoisotopic masses of all the ions in the UMC. For the peptide corresponding to the UMC, its abundance was estimated as the summation of the abundances of all the ions in the UMC (UMC intensity). The refined precursor masses of the MS/MS spectra by PE-MMR were matched to the UMC masses and then were replaced with those of UMCs. In this process, MS/MS spectra information was linked to the matched UMC. The linked MS/MS spectra were assigned with a peptide sequence (peptide ID) with an FDR ≤ 0.01 after MS-GF + searching and target-decoy analysis. The peptide ID was assigned to the UMC, and the UMC intensity was assigned to the peptide ID.

### Assignment of UMCs by a master accurate mass and time tag database

To assign the peptide IDs to unidentified UMCs, the master accurate mass and time tag (AMT) database (DB) was constructed and utilized as described previously^31^. Briefly, the information about UMCs with peptide IDs from 24 LC-MS/MS data (triplicate LC-MS/MS experiments of 8 samples) was compiled into the master AMT DB. The AMTs are unique peptide sequences whose monoisotopic masses and normalized elution times (NETs)^32^ are experimentally determined. For each AMT whose corresponding peptides were measured multiple times, the average mass and the median NET were recorded. We then mapped unidentified UMCs to AMTs in the master AMT DB with ±10 p.p.m. of mass and ±0.02 of NET tolerances. After an unidentified UMC was found to be mapped to an AMT using the mass and NET tolerances, we further computed a Xcorr measure to evaluate the similarity between their MS/MS spectra. Finally, the unidentified UMC was decided to be matched to the AMT when Xcorr was larger than the cutoff of 3.0. For each unidentified UMC matched to an AMT, the peptide ID for the AMT was assigned to the unidentified UMC, together with all information for the AMT (UMC mass and NET).

### Alignment of the identified peptides

After the assignment of unidentified UMCs using the master AMT DB, we combined the assigned and identified UMCs from LC-MS/MS datasets into an *nm* alignment table (peptide IDs for *n* UMCs and UMC intensities in *m* samples). The missing value in the alignment table means that the peptide was not identified in the corresponding datasets. For the missing values in each row of the table, we further searched for the UMCs that could be matched to the aligned UMCs based on their UMC masses and NETs with the following tolerance: ±10 p.p.m. and ±0.01 across the technical replicates, and ±10 p.p.m. and ±0.02 across the biological replicates, respectively. Quantile normalization was performed for UMC intensities in the alignment table to correct systematic variations of peptide abundances across datasets^33^. To evaluate the reproducibility of LC-MS/MS analysis, two types of similarity scores between LC-MS/MS datasets were calculated by measuring the overlap of the detected peptides and their intensity values, as previously described^34^.

### Identification of differentially secreted proteins

To identify differentially secreted proteins (DSPs), we first selected differentially expressed peptides (DEpeptides) by applying a previously reported integrative statistical method^35^ to the UMC (peptide) intensities in the alignment table. Briefly, log_2_-intensities of each peptide in the oleate-treated samples were compared to those of the untreated controls by Student’s *t*-test and the median-ratio test, which resulted in a *T* value and log_2_-median ratio, respectively, for each peptide. We then estimated empirical null distributions of *T* values and log_2_-median ratios by randomly permuting 24 samples 1000 times. For each peptide, the adjusted *P* values of the observed *T* value and log_2_-median ratio were computed using the empirical distributions with a two-tailed test and then integrated into an overall *P* value by using Stouffer’s method^36^. The DEpeptides were identified as the peptides that were detected in at least six samples for either of the conditions, with their overall *P* < 0.05 and absolute log_2_-fold-change ≥95th percentile of the empirical null distribution of log_2_-fold-changes. Moreover, we additionally selected as DEpeptides the peptides whose intensities were missing in all samples under one condition but present in at least six under the other condition. Finally, the proteins with the numbers of upregulated or downregulated unique DEpeptides ≥2 were identified as upregulated or downregulated DSPs, respectively.

### Functional enrichment analysis of detected proteins or DSPs

The enrichment analysis of Gene Ontology biological processes (GOBPs) or Kyoto Encyclopedia of Genes and Genomes (KEGG) pathways for a list of proteins (detected proteins or DSPs) was performed using DAVID software^37^. GOBPs represented by the list of proteins were identified as those with *P* < 0.01. *P* values of the processes were then converted to *Z*-scores for visualization using *Z* = -*N*^−1^(*P*), where *N*^−1^ is the inverse standard normal distribution.

### Western blotting

For western blotting, HepG2 cells were harvested in lysis buffer (10 mM Tris-HCl, pH 7.4, 1.5 mM EDTA, 10% Glycerol) containing a protease inhibitor cocktail (Roche Diagnostics, Indianapolis, USA). After sonication and centrifugation, the protein concentration was determined by the Bradford reagent assay. Finally, SDS sample buffer was mixed with lysate. Secretome samples were dissolved in lysis buffer after lyophilization to the same concentration and mixed with SDS sample buffer. The samples were separated on a 6–16% gradient SDS-PAGE gel and transferred to a nitrocellulose membrane using the Hoefer wet transfer system. After blocking with TBS-T containing 5% skim milk for 30 min, immunoblotting was performed with primary antibodies at 4 °C overnight, followed by incubation with horseradish peroxidase-conjugated anti-mouse or anti-rabbit antibody for 1 h. The membranes were washed with TBS-T buffer and developed using ECL solution. Western blotting was performed in more than four biological replicates (*n* ≥ 4) with the antibodies against PDIA3 (Abcam, Cambridge, UK), APOA1 (Abcam, Cambridge, UK), THBS1 (Thermo Fisher Scientific, MA, USA), MIF (Thermo Fisher Scientific, MA, USA), FASN (Abcam, Cambridge, UK), and EEF2 (Abcam, Cambridge, UK).

### Data availability

The mass spectrometry proteomics data have been deposited at the ProteomeXchange Consortium via the PRIDE^38^ (Dataset identifier PXD007906 and 10.6019/PXD007906).

## Results

### Secretome profiling of oleate-treated HepG2 cells

**Fig. 1:**
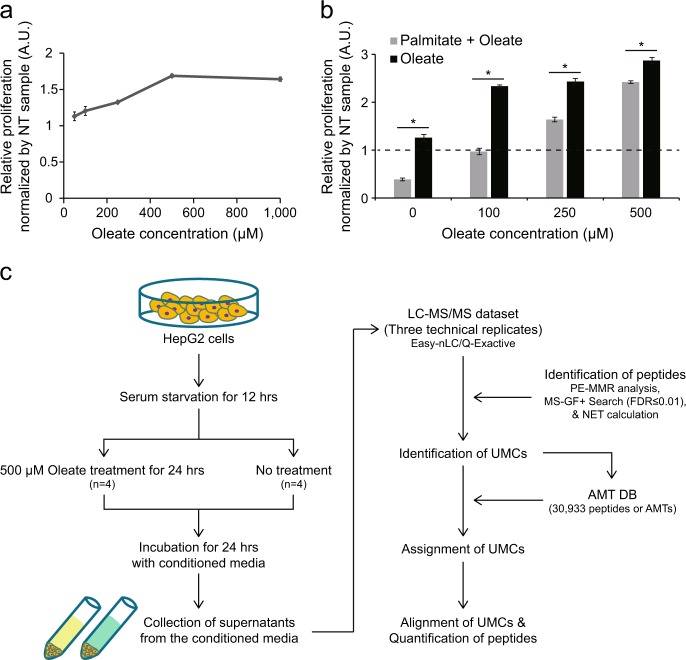
Cell proliferation assay and secretome profiling of oleate-treated and untreated HepG2 cells. **a**, **b** MTT assays for measuring proliferation of HepG2 cells after treatment with different concentrations (50, 100, 250, 500, and 1000 μM) of oleate (**a**) and after cotreatment with palmitate (500 μM) and oleate (0, 100, 250, or 500 μM) (**b**). The data are shown as the mean ± SEM (*n* = 4). *P < 0.05 by Student’s *t*-test. The dashed line denotes the threshold of relative proliferation = 1. **c** An overall scheme for the secretome profiling. HepG2 cells were subjected to serum starvation for 12 h and then treated with oleate (500 μM). Oleate-treated (*n* = 4) and untreated (*n* = 4) samples were incubated for 24 h with conditioned media, and supernatants from the conditioned media were collected for sample preparation. LC-MS/MS analyses were performed for Peptide samples from oleate-treated and untreated samples with three technical replicates. The resulting LC-MS/MS datasets were analyzed using PE-MMR analysis, MS-GF + search, and AMT DB analysis for identification, assignment, and alignment of UMCs (see text)

We first treated HepG2 cells with oleate for 24 h after serum starvation for 12 h and then measured the growth of HepG2 cells using an MTT assay. Oleate-treated HepG2 cells showed enhanced cell proliferation, compared to the untreated controls (Fig. 1a). This observation was consistent with previous findings^15,16^, despite the contradictory results in several studies, as previously discussed in the Introduction (Discussion). Previously, palmitate, the most common saturated FA, was shown to inhibit the proliferation of cancer cells by activating apoptosis^39–41^. To confirm the positive effect of oleate on the proliferation of HepG2 cells, we cotreated oleate with palmitate (500 μM) and then measured the growth of HepG2 cells. We found that oleate rescued palmitate-induced apoptosis of HepG2 cells at higher concentrations of oleate than 100 μM, which indicates an anti-apoptotic effect of oleate, thus supporting the increased proliferation of HepG2 cells by oleate (Fig. 1b). Collectively, these observations suggest that the treatment of the high concentration of oleate (500 μM) caused no significant lipotoxicity that can lead to apoptosis of HepG2 cells.

To examine secreted proteins from HepG2 that could contribute to an increased proliferation of HepG2 cells, we performed secretome profiling of HepG2 cells with and without treatment of oleate (Fig. 1c). To this end, we cultured four biological replicate samples (*n* = 4) independently prepared with and without oleate treatment. The supernatants collected from the culture media were then subjected to in-solution protein digestion^42^ (Methods and materials). For the peptide sample from each replicate in the oleate-treated and untreated conditions, we performed label-free LC-MS/MS analysis with three technical replicates (*m* = 3) as previously described^31^, resulting in a total of 24 LC-MS/MS datasets, 12 (*n* × *m* *=* 12) datasets for oleate-treated and untreated conditions, respectively (Fig. 1c and Supplementary Fig. S1). Since we analyzed the conditioned media, the resulting proteome, referred to as the ‘secretome’ in this study, can include secretory proteins, as well as proteins shed from the cell surface or proteins leaked from dying cells. However, the anti-apoptotic effect of oleate (Fig. 1b) suggests that proteins leaked from apoptotic cells are unlikely to be enriched in our secretome (Discussion).

Next, peptide identification was performed for 24 LC-MS/MS datasets using PE-MMR analysis followed by an MS-GF + search with the target-decoy strategy (FDR ≤ 0.01; Supplementary Table S1a). All identified UMCs (peptides) from the 24 datasets were used to construct the master AMT DB that comprised 30,933 peptides (or AMTs; Supplementary Table S2). Using the AMT DB, unidentified UMCs in the individual samples were matched with the peptides in the DB using the mass and the NET tolerances (Methods and materials) and then assigned with IDs of the matched peptides (Supplementary Table S1b). After the AMT DB search, the peptides were quantified as their UMC intensities in the 24 LC-MS/MS datasets and then compiled into an alignment table (Fig. 1c; Methods and materials). Using peptide abundances (UMC intensities) normalized by quantile normalization^33^, we first examined the reproducibility of our label-free LC-MS/MS analysis using the ID and intensity similarity scores described by Mueller et al^34^. The average ID and intensity similarity scores in the 24 LC-MS/MS datasets were found to be 0.94 and 0.90, respectively, suggesting high reproducibility of the label-free LC-MS/MS analysis (Supplementary Fig. S2). Based on the 30,933 peptides in the AMT DB, we identified 2640 HepG2 secretory proteins (2630 protein-coding genes) with high confidence as the proteins with two or more sibling unique peptides (Supplementary Table S3).

### Comparison of the measured secretome with previous secretomes

**Fig. 2:**
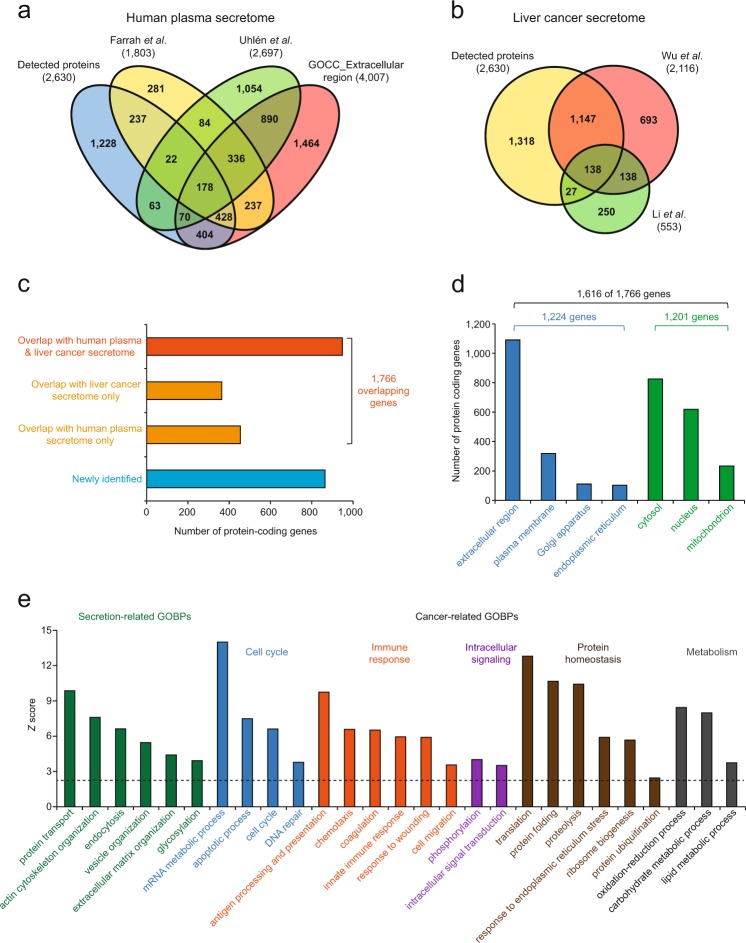
Comparison of our secretome with the previous secretomes and localization and functional analysis of the secretome. **a** Comparison of our secretome with human plasma secretomes previously reported and with protein-coding genes known to be localized in the extracellular region based on their GOCCs. **b** Comparison of our secretome with two previously reported liver cancer secretomes. **c** 1766 secreted proteins overlapped with human plasma or liver cancer secretomes. **d** Distribution of cellular localizations (GOCCs) of the overlapping secreted proteins. Bar colors represent the numbers of secreted proteins localized in the compartments related (blue) or unrelated (green) to secretion. **e** Cellular processes (GOBPs) represented by the 1766 overlapping secreted proteins. Bar colors indicate a group of the process related to secretion (green) and five groups of the processes related to cancer pathophysiology (blue for cell cycle, orange for immune response, purple for intracellular signaling, brown for protein homeostasis, and gray for metabolism). The dashed line denotes the cutoff of *P* = 0.01

To assess the comprehensiveness of our secretome, we compared the 2630 detected protein-coding genes with (1) two human plasma secretomes previously reported^43,44^ and (2) the 4007 genes whose cellular localization was annotated with ‘extracellular region’ based on Gene Ontology cellular compartments (GOCCs) (Fig. 2a). The two human plasma secretomes reported by Farrah et al^43^. and Uhlén et al^44^. included the proteins encoded from 1803 and 2697 genes, respectively. Of the 2630 protein-coding genes in our secretome, 1402 (2630−1228 = 1402 in Fig. 2a; 53.3%) were detected in at least one of the two plasma secretomes or annotated with an extracellular region based on GOCCs. Next, we further compared our secretome with two liver cancer secretomes previously reported^4,45^ (Fig. 2b). Wu et al^4^. profiled the secretomes of three liver cancer cell lines (HepG2, Hep3B, and SK-Hep-1), which included 2116 protein-coding genes. Additionally, Li et al^45^. profiled the N-glyco-secretomes of two liver cancer cell lines (MHCC97-L and HCCLM3), which included 553 protein-coding genes. Of the 2630 protein-coding genes in our secretome, 1312 genes (2630–1318 = 1312 in Fig. 2b; 49.9%) were shared with the two liver cancer secretomes. Taken together, of the 2630 protein-coding genes in our secretome, 1766 genes (67.1%) were detected in either the human plasma or the liver cancer secretomes (Fig. 2c), which supports the validity of our secretome. The remaining 864 nonoverlapping genes were newly identified in our study as the ones whose protein products are to be secreted (Fig. 2c), which suggests the comprehensiveness of our secretome.

Next, we examined GOCCs enriched by the 1766 overlapping protein-encoding genes using DAVID software^37^. Of the 1766 overlapping genes, 1224 were localized in secretion-related GOCCs, such as the extracellular region, plasma membrane, Golgi apparatus, and endoplasmic reticulum (ER; Fig. 2d). Interestingly, on the other hand, a significant number (1201 genes) of the 1766 overlapping genes were previously reported to be localized in the cytosol, nucleus, or mitochondrion. These data suggest a hypothesis that our secretome may include the proteins in microvesicles released from HepG2 cells. In support of this hypothesis, the 1201 genes showed a significant (57.6%) overlap with the exosome proteome annotated by GOCCs (Supplementary Fig. S3). Moreover, to examine cellular processes associated with the 1766 overlapping genes, we performed the enrichment analysis of GOBPs for the 1766 genes using DAVID software (Fig. 2e and Supplementary Table S4). The overlapping genes were significantly (*P* < 0.01) associated with secretion-related processes (protein transport, extracellular matrix organization, vesicle organization, actin cytoskeleton organization, and glycosylation), as well as various cancer-related processes including cell cycle (cell cycle, DNA repair, apoptotic process, and mRNA metabolic process), immune responses (antigen processing and presentation, chemotaxis, coagulation, innate immune response, and cell migration), intracellular signaling (phosphorylation and intracellular signal transduction), protein homeostasis (translation, ribosome biogenesis, protein folding, and protein ubiquitination), and metabolism (carbohydrate and lipid metabolic processes). These data suggest that our secretome includes secreted proteins that can contribute to modulating the processes related to cancer pathogenesis.

### Secretome alterations by oleate treatment and its associated processes

To determine secreted proteins whose abundances were altered in HepG2 cells by oleate treatment, we first aligned the 30,933 quantified peptides in the 24 LC-MS/MS datasets (12 oleate-treated and 12 untreated samples) and then compared their abundances between oleate-treated and untreated samples (Methods and materials). For the aligned peptides, we identified 2461 unique peptides (DEpeptides) showing significant (*P* < 0.05) upregulation or downregulation by oleate treatment using the integrative statistical method previously reported^35^ (Methods and materials). We then identified 349 DSPs that have two or more DEpeptides showing consistent upregulation or downregulation by oleate treatment as previously described^46^ (Methods and materials). We excluded 12 DSPs having both two or more upregulated and downregulated DEpeptides. Of the remaining DSPs, 145 were upregulated by oleate treatment, while 192 were downregulated (Supplementary Table S5).

**Fig. 3:**
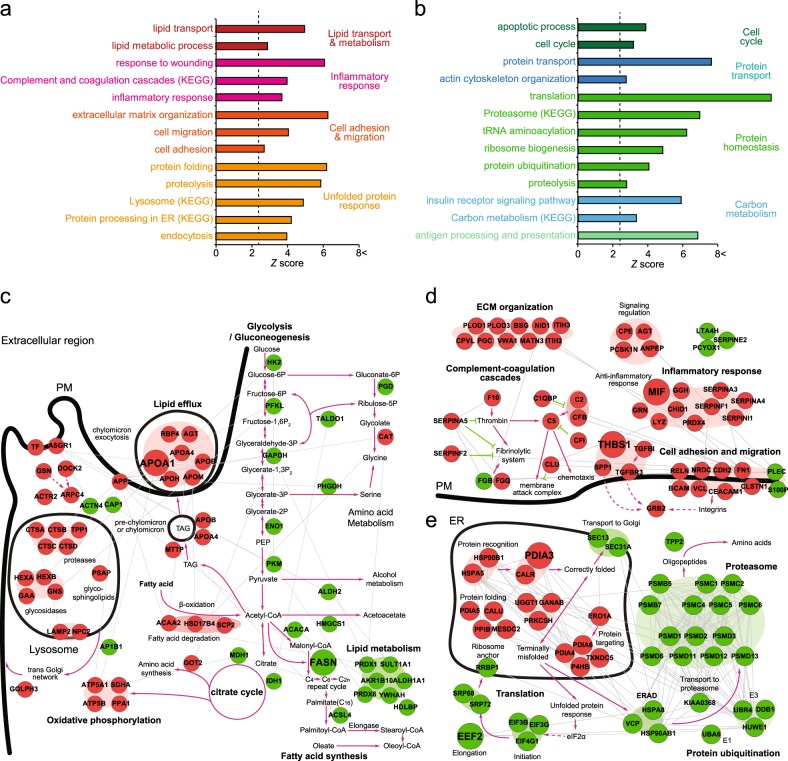
Cellular processes represented by DSPs between oleate-treated and untreated samples and network models delineating key cellular processes. **a**, **b** Cellular processes (GOBPs) enriched by upregulated (**a**) and downregulated (**b**) secreted proteins between oleate-treated and untreated samples. Each bar represents the *Z*-score of the *P* value that is the significance of the corresponding GOBP being enriched by the DSPs. The dashed line denotes the cutoff of *P* = 0.01. Bar colors represent the groups of GOBPs associated with the indicated cellular events. **c–e** Network models describing interactions among DSPs involved in FA removal and production (**c**), inflammatory responses and cell adhesion/migration (**d**), and ER stress and protein degradation (**e**). Node colors represent up- (red) and down-regulation (green) of secreted proteins after oleate treatment. Large nodes indicate the six secreted proteins selected as a secretome profile in this study. Nodes were arranged according to GOCCs and the localization information obtained from the Kyoto Encyclopedia of Genes and Genomes (KEGG) pathway database. Gray lines represent protein-protein interactions, and the *arrows* and *inhibition symbols* obtained from the KEGG pathway database denote activation and suppression, respectively. Thick black lines denote membranes, such as plasma membrane (PM), lysosomal (**c**) and ER (**e**) membranes, and vesicular membranes (**c**)

To understand cellular functions associated with the DSPs, the enrichment analyses of GOBPs and KEGG pathways were performed for 145 upregulated and 192 downregulated secreted proteins using DAVID software. The upregulated secreted proteins by oleate treatment were significantly (*P* < 0.01) associated with the following processes (Fig. 3a and Supplementary Table S6a): (i) lipid transport and metabolism, (ii) inflammatory response (complement and coagulation cascades, inflammatory response, and response to wounding), (iii) cell adhesion and migration (cell adhesion, cell migration, and extracellular matrix organization), and (iv) unfolded protein response (protein processing in ER, protein folding, proteolysis, lysosome, and endocytosis). On the other hand, the downregulated secreted proteins by oleate treatment were associated with the following processes (Fig. 3b and Supplementary Table S6b): (i) cell cycle (cell cycle and apoptosis process), (ii) protein transport (protein transport and actin cytoskeleton organization), (iii) protein homeostasis (translation, tRNA aminoacylation, ribosome biogenesis, protein ubiquitination, proteasome, and proteolysis), (iv) carbon metabolism (insulin receptor signaling and carbon metabolism), and (v) antigen processing and presentation.

### Cellular networks associated with the secretome altered by oleate treatment

To understand the functions of the cellular processes associated with upregulated and downregulated secreted proteins, we next reconstructed a network model describing the interactions among the DSPs involved in their associated GOBPs mentioned above (Methods and materials). The network model first showed upregulation of the following metabolic pathways contributing to FA removal: (1) fatty acid degradation (ACAA2, HSD17B4, and SCP2), (2) incorporation of triglyceride (TAG) into pre-chylomicrons (MTTP, APOA4, and APOB), and (3) chylomicron exocytosis or lipid efflux (RBP4, APOA1, APOA4, APOB, APOH, and APOM) (Fig. 3c, center). In contrast, it showed downregulation of the following metabolic pathways contributing to FA production: (1) FA synthesis (FASN, ACACA, and ACSL4), as well as (2) glycolysis (HK2, PFKL, GAPDH, ENO1, and PKM) and (3) the citrate cycle (MDH1 and IDH1), which contribute to the generation of acetyl CoA, the precursor of FA synthesis (Fig. 3c, right). Additionally, upregulation of the metabolic pathways branching from the citrate cycle for amino acid synthesis and oxidative phosphorylation also contribute to the reduction of metabolic fluxes for the generation of acetyl-CoA. In addition, the network model showed upregulation of lysosomal pathways involved in lipophagy to remove lipid droplets formed by free FA and TAG (Fig. 3c, left). All these alterations in the secretome reflect that oleate treatment increases the intracellular FA amount in HepG2 cells and thereby modulates metabolic pathways to reduce the FA amount through increased FA removal and decreased FA production. Moreover, the network model showed upregulation of inflammatory responses, including the complement and coagulation pathway and neutrophil or platelet degranulation (Fig. 3d, left). Additionally, it showed upregulation of ECM organization and cell adhesion and migration (Fig. 3d, right). These alterations in the secretome suggest that oleate treatment can contribute to inflammatory responses, as well as cell adhesion and migration. Finally, the network model showed upregulation of the unfolded protein response pathway in the ER, consistent with the previous finding that a nutrient excess derived by oleate treatment contributes to the activation of ER stress^47,48^ (Fig. 3e, left). In contrast, protein ubiquitination and proteasome activities were downregulated (Fig. 3e, right). These alterations in the secretome suggest that oleate treatment can result in increased ER stress but decreased protein degradation.

### A secretome profile associated with oleate-induced proliferation of HepG2 cells

The network models above showed that inflammatory responses (Fig. 3d), ER stress (Fig. 3e), and lipid metabolism (Fig. 3c) were associated with the altered secretome by oleate treatment. Previously, a number of studies have reported associations of these processes with cell proliferation. Thus, to select a secretome profile that can represent oleate-induced proliferation of HepG2 cells (Fig. 1a), we focused on the DSPs involved in these processes. First, for inflammatory response, oleate was shown to induce anti-inflammatory responses^49,50^. The network model showed an upregulation of molecules involved in the anti-inflammatory response. Thus, we selected the following representative secretome proteins for the anti-inflammatory response in the network model (Fig. 3d): (1) macrophage migration inhibitory factor (MIF) and (2) thrombospondin 1 (THBS1). MIF inhibits infiltration of macrophages^51^, and THBS1 is secreted and promotes the resolution of inflammation^52^.

Second, nutrient starvation^53,54^ or excess^47,48^ in tumor cells induces ER stress, which is the main site for translation of nutrition into metabolic and inflammatory responses. A mild ER stress response is considered cytoprotective, which is often accompanied by altered translation, contributing to tumor survival and adaptation against harsh environments^55–57^. In our secretome, ER stress response was upregulated together with downregulated translation (Fig. 3e). Thus, as the representative DSPs for ER stress response-related processes in the network model, we selected protein disulfide isomerase family A member 3 (PDIA3) involved in the ER stress-induced UPR response and eukaryotic translation elongation factor 2 (EEF2) involved in translation (Fig. 3e). Finally, in highly proliferative tumor cells, lipid-based metabolic reprogramming is important for the supply of energy and biomass components^58^. Lipid homeostasis (lipid transport, biosynthesis, and degradation) has been considered to be closely associated with the proliferation of tumor cells^59,60^. In our secretome, lipid transport and degradation were upregulated by oleate treatment, while lipid biosynthesis was downregulated. Thus, as the representative DSPs for oleate-induced alterations in lipid metabolism, we selected apolipoprotein A1 (APOA1) involved in lipid efflux and fatty acid synthase (FASN) involved in lipid biosynthesis (Fig. 3c). In total, the six proteins mentioned above were selected as a secretome profile that can be associated with oleate-induced proliferation of tumor cells.

**Fig. 4:**
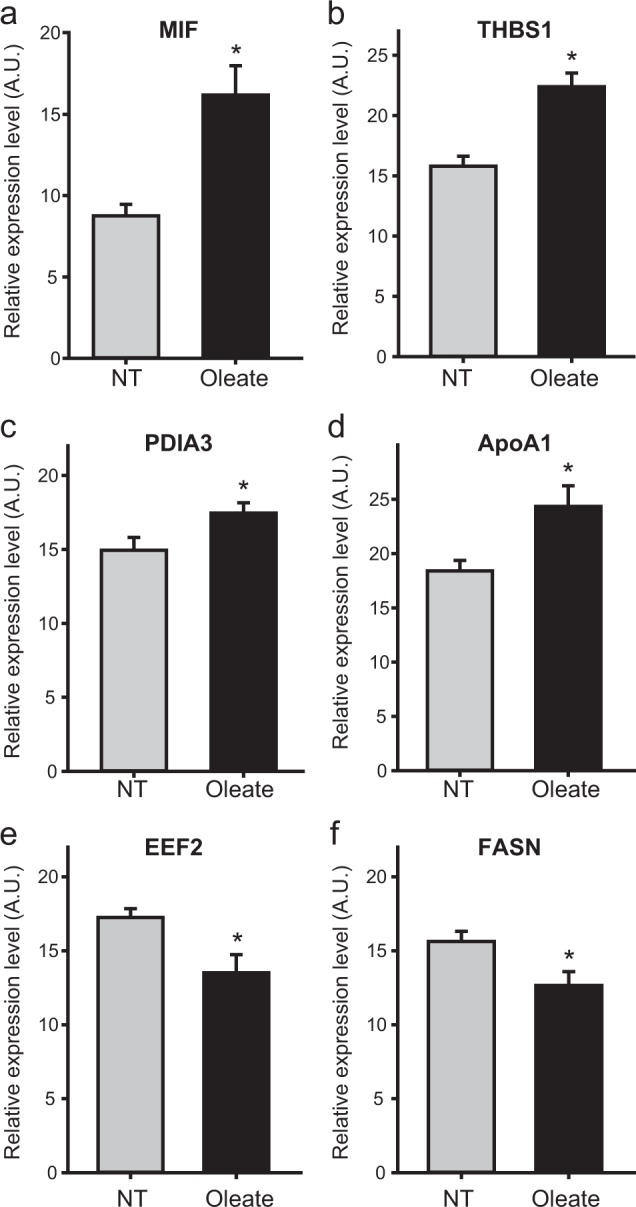
Validation of upregulation and downregulation of the selected secreted proteins after oleate treatment. **a–f** Western blotting analysis of the six selected secreted proteins using an independent set of the conditioned media samples (*n* ≥ 4): MIF (**a**), THBS1 (**b**), PDIA3 (**c**), APOA1 (**d**), EEF2 (**e**), and FASN (**f**). The results showed significant (*P* < 0.05) upregulation of MIF, THBS1, PDIA3, and APOA1 and downregulation of EEF2 and FASN in oleate-treated samples compared to the untreated controls. The data are shown as the mean ± SEM. *P < 0.05 by Student’s *t*-test

To test the validity of the six selected proteins, we cultured the samples independently prepared with and without oleate treatment and collected the supernatants from the culture media. In these new samples, using western blotting, we then tested the upregulation or downregulation of the selected secreted proteins. The results showed that MIF, THBS1, PDIA3, and APOA1 were upregulated (Fig. 4a–d) and FASN and EEF2 were downregulated (Fig. 4e–f) in oleate-treated samples, compared to untreated samples, confirming their differential expression measured by LC-MS/MS analysis after oleate treatment. These data indicate that the six secreted proteins can be used as a secretome profile that represents the oleate-induced proliferation of tumor cells.

## Discussion

In liver cancer, the increased FA is a key feature of highly proliferative tumors due to cancer-related metabolic alterations. The secreted factors by the increased FA can modulate the proliferation of tumor cells. A panel of secreted factors can be used to assess FA-driven metabolic reprogramming for tumor survival. However, the secreted factors affected by the increased FA have not been systematically explored. In this study, we examined comprehensive secretome profiles affected by the increased FA and identified a secretome profile representing FA-induced proliferation of tumor cells. To achieve this goal, we employed an approach that involves (1) comprehensive secretome profiling of a culture medium of HepG2 cells with and without oleate treatment; (2) identification of DSPs between oleate-treated and untreated samples; (3) selection of a secretome profile that can represent cellular processes (lipid metabolism, inflammatory response, and ER stress) associated with proliferation of tumor cells; and (4) validation of the selected secretome profile in independent samples using western blotting. Using this approach, we identified a secretome profile composed of six DSPs by oleate treatment (MIF, THBS1, PDIA3, EEF2, APOA1, and FASN).

In this study, we found that oleate treatment promoted the proliferation of HepG2 cells. However, there have been conflicting reports regarding the effect of oleate on the proliferation of HepG2 cells. Several studies showed that oleate treatment led to the promotion of proliferation of HepG2 cells^15,16^, but other studies reported that oleate treatment reduced the proliferation of HepG2 cells and induced apoptosis of HepG2 cells^61^. These contradictory observations in these studies might be due to differences in culture conditions, doses of oleate used, or states of the cells. Moreover, the two previous studies mentioned above^15,16^ showed that the high concentration of oleate (500 µM) used in this study induced apoptosis of hepatoma cell lines. It can thus be speculated that the secretome measured after oleate treatment may include a mix of real secretion and necro-apoptotic content from HepG2 cells. However, our further experiments showed that palmitate-induced apoptosis of HepG2 cells was rescued by oleate treatment, which supports the promotion of proliferation of HepG2 cells by oleate treatment. In addition, LC-MS/MS analysis measured caspases 3 and 7, which are marker proteins for apoptosis, from HepG2 cells with and without the treatment of oleate. We found that the peptides from these caspases showed no significant differences in their abundances between the oleate-treated cells and untreated controls (Supplementary Fig. S4a). Moreover, due to the anti-apoptotic effect of oleate, proportions of the proteins previously reported to be localized in nonsecretory organelles (cytosol, nucleus, or mitochondria) can be reduced in oleate-treated conditions, compared to that in untreated conditions. We thus examined the proportion of proteins in nonsecretory organelles among 2607 and 2615 protein-coding genes detected from oleate-treated and untreated conditions, respectively, and found that the proportions (67.9%) were similar between the oleate-treated and untreated conditions (Supplementary Fig. S4b). However, the effect of oleate on proteins leaked from dying cells can be reflected in their abundances beyond whether they were detected or not. Thus, we further examined the proportions of DSPs and non-DSPs in nonsecretory organelles by the oleate treatment and found that the proportions were similar between DSPs (69.0%) and non-DSPs (67.5%) (Supplementary Fig. S4c). All of these data suggest that no significant apoptosis was induced by oleate and thus no significant contamination of the secretome with the apoptotic content.

We showed that lipid metabolism, inflammatory response, and ER stress were upregulated in the secretome of the oleate-treated HepG2 cells. Previously, the interplays among these processes have been reported under pathological conditions, including cardiovascular diseases and cancers. For example, the interplay between lipid metabolism and inflammation in metabolic tissues can aggravate the development of atherosclerosis^62^. Saturated FAs induce pro-inflammatory signaling, while unsaturated FAs (omega-3 and omega-9 FAs) induce anti-inflammatory signaling^62^. In particular, oleate was shown to induce anti-inflammatory responses^49,50^. This anti-inflammatory effect of oleate is consistent with upregulation of molecules associated with the anti-inflammatory response (Fig. 3d) in our secretome. Moreover, the interplay between lipid metabolism and ER stress was previously observed in hepatic steatosis^63^. Similarly, saturated FAs activated ER stress leading to apoptosis in steatotic rat liver, but unsaturated FAs (oleate and linoleic acid) rescued palmitate-induced ER stress and apoptosis^64^. This observation is consistent with the upregulation of cytoprotective mild ER stress (ER network in Fig. 3e) in our secretome. Thus, our secretome data suggest that oleate can induce the interplays among these three processes within HepG2 cells. However, the inference of intracellular regulatory pathways underlying the interplays among these processes still remains challenging due to limited information that can be used to predict the intracellular pathways in our secretome data. Nevertheless, the network models revealed an upregulation of transcription and signaling regulators related to lipid metabolism (RBP4, AGT, and APOA1), inflammation (SPP1, TGFBR3, and THBS1), and ER stress (HSP90B1, PDIA3, and PRKCSH), which were upregulated in the secretome of oleate-treated HepG2 cells, suggesting their potential roles in the interplays among these processes, which can be tested in detailed functional experiments.

A number of previous studies have shown associations of the proteins selected in this study, especially the four upregulated proteins (MIF, THBS1, PDIA3, and APOA1), with various features of cancer pathophysiology in liver cancer, as well as other types of cancers (Supplementary Table S7): (1) MIF was reported to be increased at both the mRNA and protein levels in liver cancer tissues compared to noncancerous tissues^65^ and to promote tumor survival, angiogenesis, and/or metastasis in colon, head and neck, liver, and/or prostate cancers;^65–68^ (2) THBS1 was shown to be elevated in liver cancer^69^ and to promote tumor survival, aggressiveness, angiogenesis, and metastasis in breast, bladder, liver, gastric, prostate, and/or pancreatic cancers;^69–74^ (3) PDIA3 was reported to be elevated in liver cancer^75^ and to enhance the proliferation of tumor cells, metastasis, and/or invasion in breast, colon, ovarian, and/or pancreatic cancers;^75–79^ and (4) APOA1 was observed to be elevated in the serum of liver cancer patients^80^, as well as in the serum of breast, colon, and lung cancers^81–83^ and in the urine of bladder cancer^84^, and its abundance showed positive correlations with aggressiveness and/or metastasis in bladder, colon, and lung cancers^82,85,86^. These data suggest that the secreted proteins with various tumors might serve as a secretome profile that can represent cancer pathophysiology, such as the proliferation of tumor cells. However, none of these secreted proteins have been previously reported to be altered in their protein levels by the increased FA.

Several proteomic studies have provided the secretomes of various cancers and/or lists of DSPs in cancers compared to controls (Supplementary Table S8). First, Wu et al^4^. provided the secretome of 23 cancer cell lines derived from 11 types of cancers, which included 4584 non-redundant proteins, and then proposed pan-cancer markers and also selective markers for each type of cancer. In addition, Planque et al^87^. profiled the secretome of lung cancer cell lines, including 1830 secretory proteins, and proposed a set of biomarker proteins for lung cancer. Moreover, Ralhan et al^88^. identified 122 secreted proteins from head and neck cancer cell lines; Sardana et al^3^. identified 2124 secreted proteins from prostate cancer cell lines; and Gunawardana et al^89^. identified 420 secreted proteins from ovarian cancer cell lines. Second, a number of studies have performed comparative secretome analyses of tumor and normal samples in various types of cancers, including breast, colon, esophagus, gastric, head and neck, liver, and pancreatic tumors, providing the lists of DSPs in these cancer samples compared to normal controls (Supplementary Table S8). The selected proteins in this study were detected in many of these previous secretome studies and further identified as DSPs in one of the previous studies. However, their upregulation or downregulation patterns were different depending on the type of cancer and cell lines used and/or culture conditions, suggesting that their abundances could be used as indicators of different conditions in the tumor microenvironment. Nevertheless, none of the selected proteins have been previously reported to be altered in their secretion by increased oleate.

Our study showed differential secretion of the six selected proteins, which have been previously reported to be associated with the proliferation of tumor cells, thus supporting their potential use as an indicator of FA-induced proliferation of tumor cells. The clinical implications of these selected proteins can be tested with a larger number of tumor patients. In addition, longitudinal studies can be designed to further demonstrate the nature of the altered secretion of the selected proteins during the course of tumorigenesis. Additionally, novel subtypes of liver cancer patients might be further characterized based on the status of the FA-induced proliferation of tumor cells or the FA-induced metabolic reprogramming represented by the proposed secretome profile. In addition to the selected proteins, our approach provided a comprehensive list of DSPs associated with lipid metabolism, inflammatory response, and ER stress, thus extensively extending the current list of tumor survival-related secreted proteins identified by conventional small-scale experiments or approaches. This list of secreted proteins can serve as comprehensive resource to biologists who study FA-dependent tumor survival. Furthermore, the network models can provide the basis for an understanding of the roles of secreted proteins in the FA-induced metabolic reprogramming (Fig. 3c), inflammatory response (Fig. 3d), and ER stress (Fig. 3e) in tumor survival. The network models further suggested that lysosomal activation (Fig. 3c) and protein degradation (Fig. 3e) associated with the DSPs could be involved in tumor survival. This understanding can further suggest proactive therapeutic strategies for FA-induced tumor survival. In summary, our approach successfully identified a secretome profile that can provide a novel dimension of the information indicative of FA-induced tumor survival for classification, therapy, and pathogenesis of liver cancer.

## Electronic supplementary material

Supplementary Information

Supplementary Table S2

Supplementary Table S3

Supplementary Table S4

Supplementary Table S5

Supplementary Table S6

Supplementary Table S7

Supplementary Table S8
