# Supplementary material for: A secretome profile indicative of oleate-induced proliferation of HepG2 hepatocellular carcinoma cells
Source: Exp Mol Med. 2018 Aug 3;50(8):93. doi: 10.1038/s12276-018-0120-3 (PMC6076227; doi:10.1038/s12276-018-0120-3)
Supplement: Supplementary file 1 — Supplementary Information [file 12276_2018_120_MOESM1_ESM.docx]

**A secretome profile indicative of oleate-induced proliferation of HepG2 hepatocellular carcinoma cells**

Soyeon Park1,*, Ji-Hwan Park2,*, Hee-Jung Jung2,*, Jin-Hyeok Jang3, Sanghyun Ahn2, Younah Kim2, Pann-Ghill Suh4, Sehyun Chae2, Jong Hyuk Yoon1,5,#, Sung Ho Ryu1,#, and Daehee Hwang2,6,#

1Department of Life Sciences, Pohang University of Science and Technology (POSTECH), Pohang, Kyungbuk, 37673, Republic of Korea; 2Center for Plant Aging Research, Institute for Basic Science (IBS), Daegu, 42988, Republic of Korea; 3Department of Brain and Cognitive Sciences, Gyeongbuk Institute of Science and Technology (DGIST), Daegu, 42988, Republic of Korea; 4School of Life Sciences, Ulsan National Institute of Science and Technology, Ulsan, 44919, Republic of Korea; 5Korea Brain Research Institute, Daegu, 41068, Republic of Korea; and 6Department of New Biology, DGIST, Daegu, 42988, Republic of Korea;

*These authors have contributed equally to this work.

#Corresponding authors :

Daehee Hwang, Ph.D.,

Center for Plant Aging Research, Institute for Basic Science (IBS), and Department of New Biology, Daegu Gyeongbuk Institute of Science and Technology (DGIST), Daegu, 42988, Republic of Korea,

Phone: 82-53-785-1840; Fax: 82-53-785-1809; E-mail: [dhwang@dgist.ac.kr](mailto:dhwang@dgist.ac.kr)

Sung Ho Ryu, Ph.D.,

Department of Life Sciences, Pohang University of Science and Technology (POSTECH), Pohang, Kyungbuk, 37673, Republic of Korea

Phone: 82-54-279-2292; Fax: 82-54-279-0645; E-mail: [sungho@postech.ac.kr](mailto:sungho@postech.ac.kr)

Jong Hyuk Yoon, Ph.D.,

Department of Neural Development and Disease, Korea Brain Research Institute, Daegu, 41068, Republic of Korea

Phone: 82-53-980-8341; Fax: 82-53-980-8399; E-mail: [jhyoon@kbri.re.kr](mailto:jhyoon@kbri.re.kr)

This file includes:

Supplementary Figures S1–4

Supplementary Tables S1–8

– Supplementary Tables S2–8 are provided in additional Excel spreadsheets.

**Supplementary Figure S1.** Base peak chromatograms of triplicate LC-MS/MS experiments for four biological replicates in oleate-treated (Ole-CM#) and untreated (NT-CM#) conditions. For each LC-MS/MS dataset, base peak chromatogram and pep3D image are shown together with the number of peptides identified from MS-GF+ search.


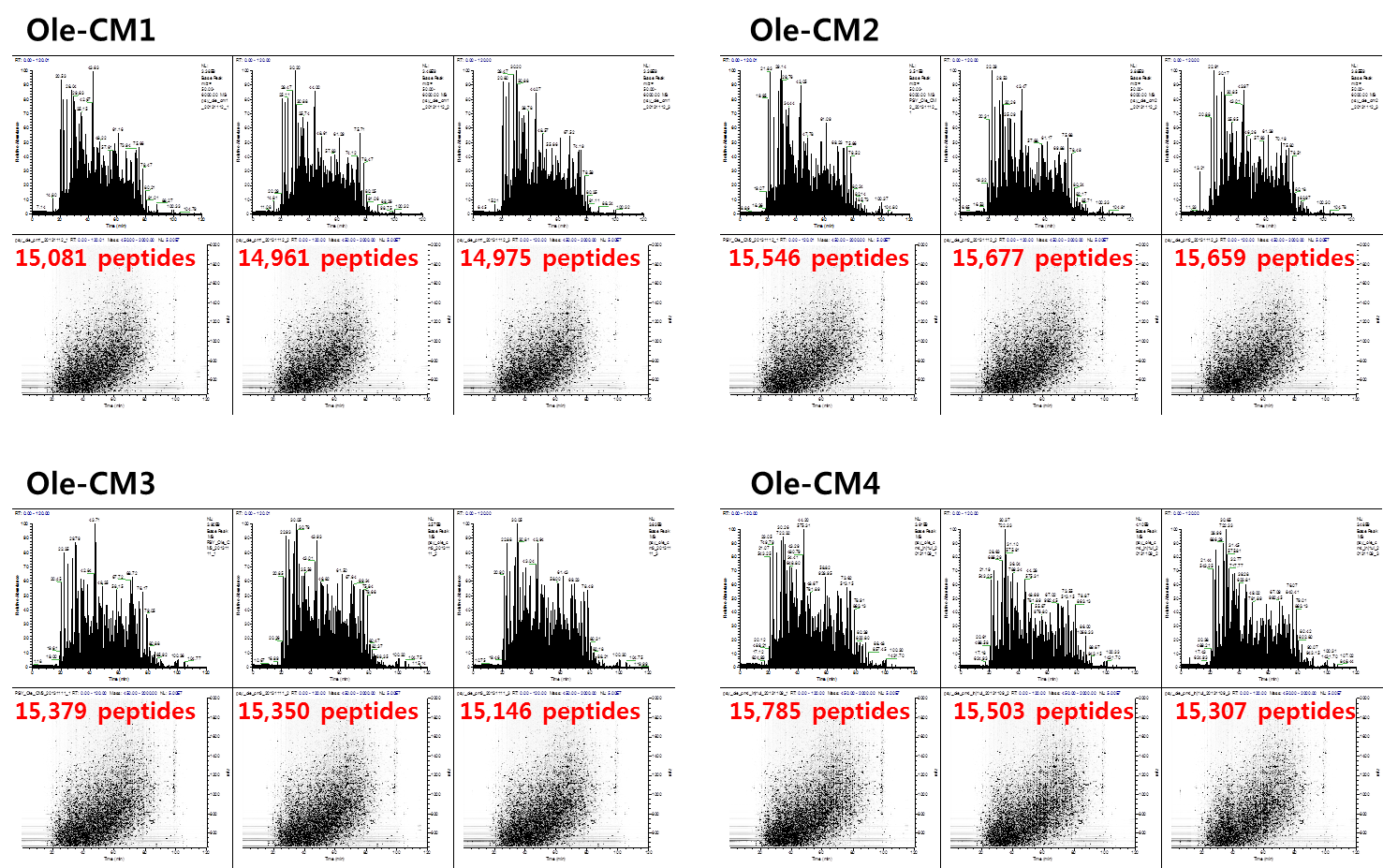


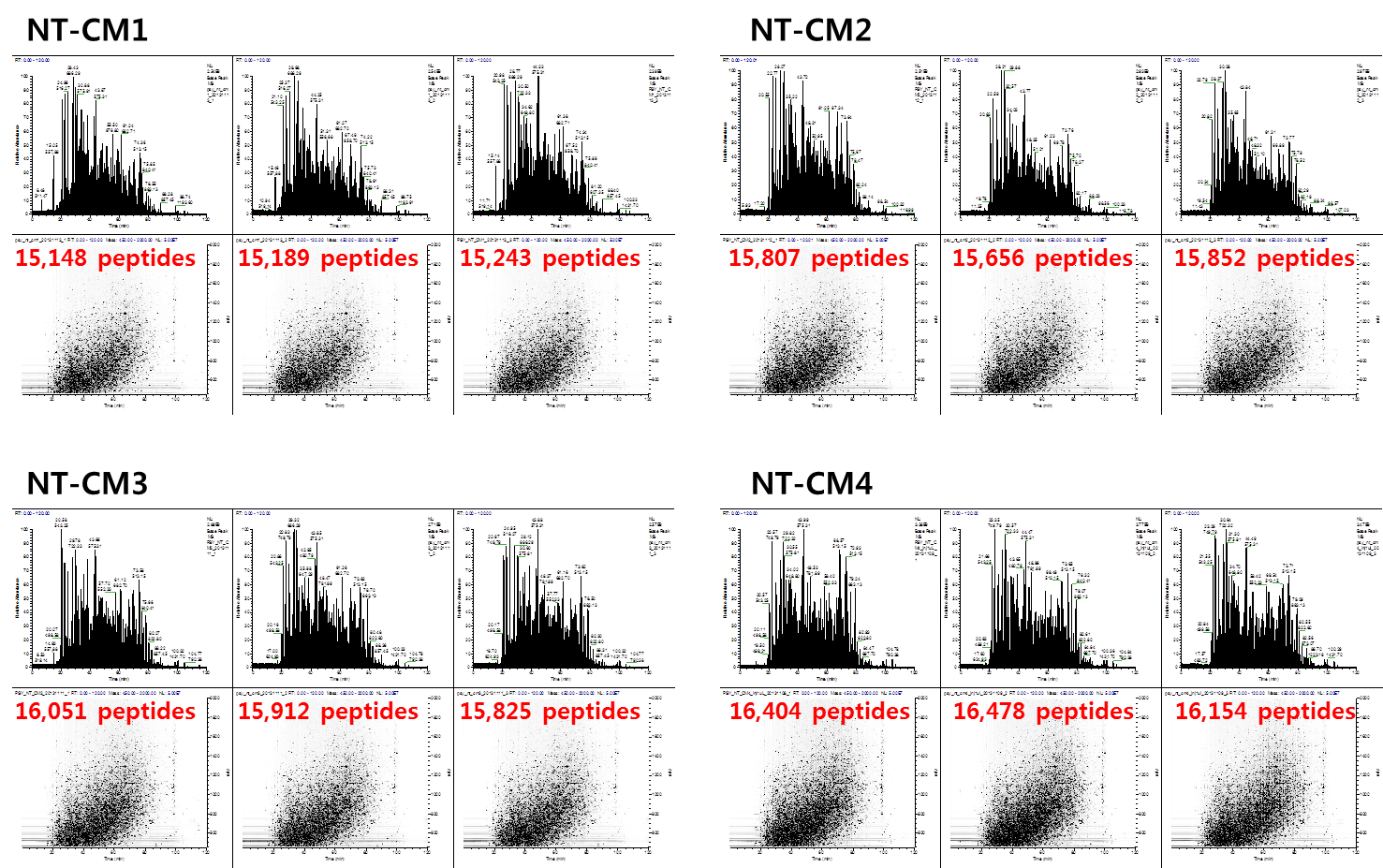


**Supplementary Figure S2.** Similarity scores among 24 LC-MS/MS datasets. (**a**, **b**) Heat maps showing ID (**a**) and intensity (**b**) similarity scores for all pairs of the 24 LC-MS/MS datasets. Four biological replicates were denoted as Rep1-4, respectively, in oleate-treated and untreated conditions (‘Oleate treated’ and ‘Control’, respectively). The color bar represents the gradient of similarity scores.


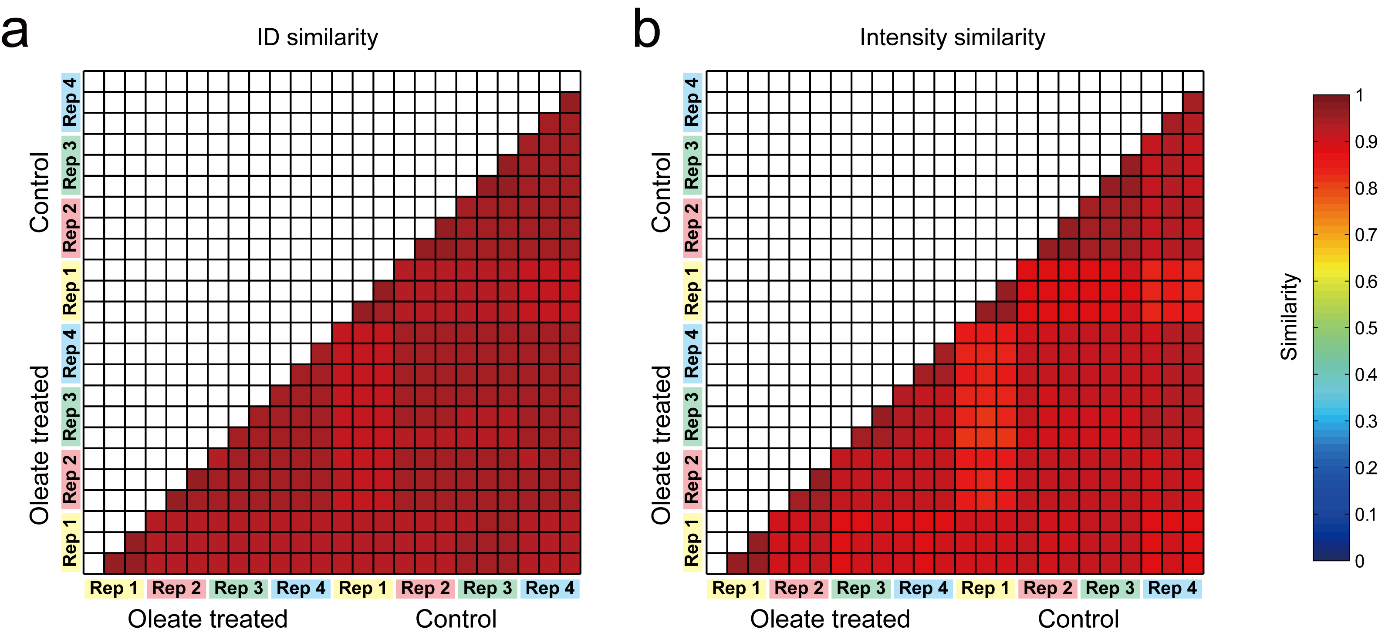


**Supplementary Figure S3.** Venn diagram of the genes localized in cytosol, nucleus and mitochondrion versus the genes localized in extracellular exosome among the 1,766 secreted protein-encoding genes overlapping with those detected in the previous secretome studies.


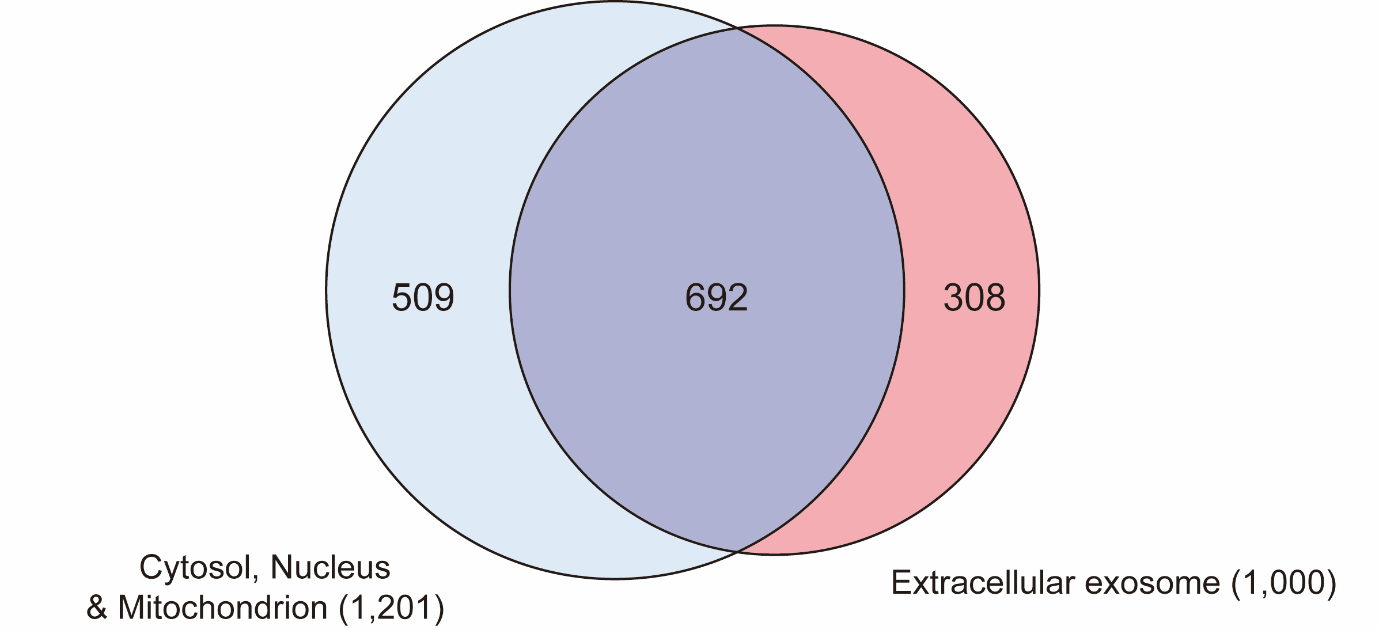


**Supplementary Figure S4.** Assessment of potential contamination of the secretome with proteins leaked from apoptotic cells. (**a**) log_2_-intensities of unique sibling peptides of caspase 3 and 7 measured in oleate-treated and untreated conditions by LC-MS/MS analysis. Values are means ± SD. (**b, c**) Comparison of proportions of the proteins previously reported to be localized in non-secretory organelles (cytosol, nucleus, or mitochondria; **Figure 2d**) between oleate-treated and untreated conditions (**b**) and between DSPs and non-DSPs (**c**).


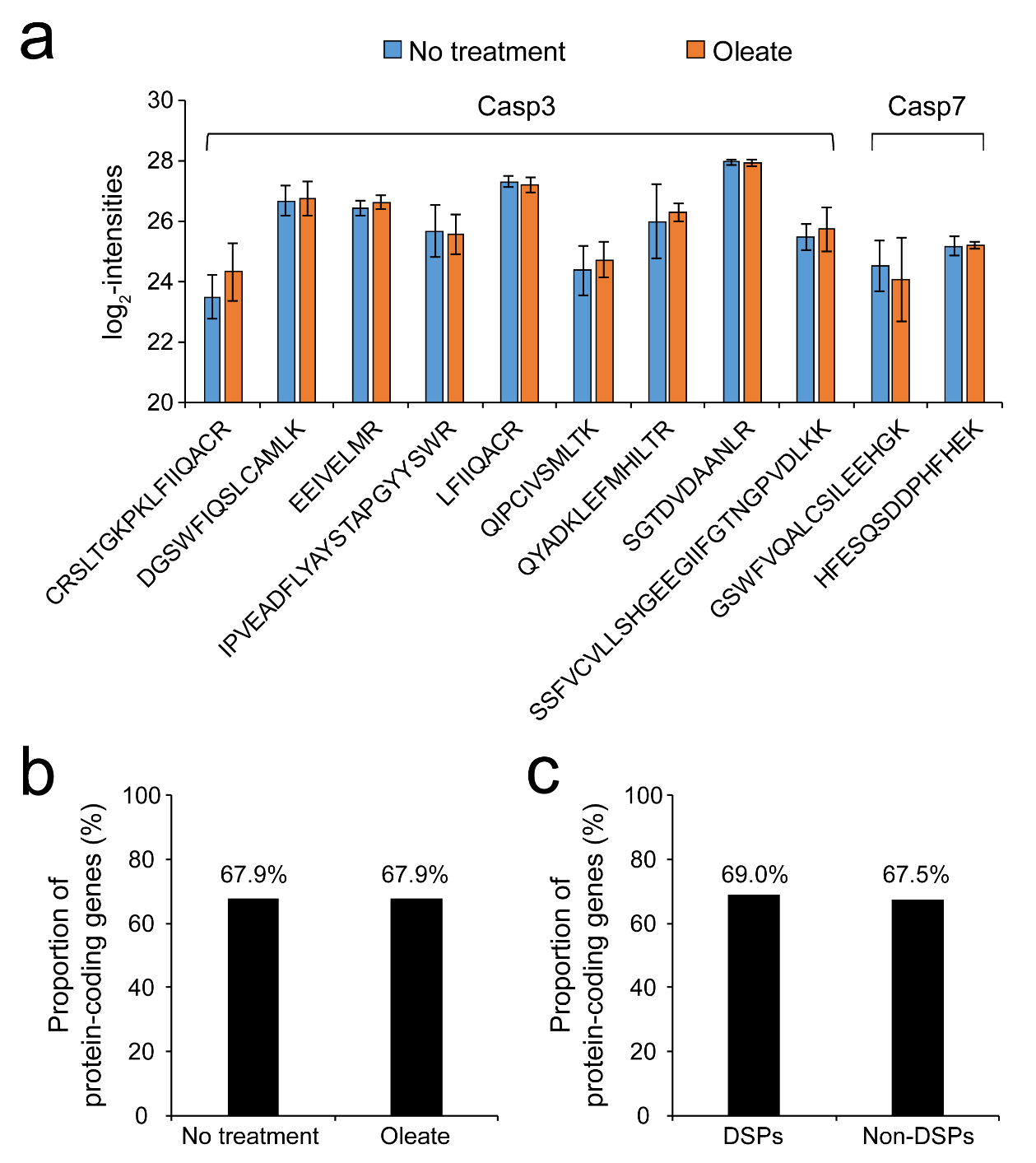


**Supplementary Table S1**. MS-GF+ and AMT-DB search.

(**a**) MS-GF+ search results. Rows represent four biological replicates in oleate-treated (Ole-CM#) and untreated (NT-CM#) conditions, and columns represent technical triplicates (1^st^, 2^nd^, and 3^rd^) for each biological replicate.

| **Replicates** | **1^st^** | **2^nd^** | **3^rd^** | **Union** |
| --- | --- | --- | --- | --- |
| **Ole-CM1** | 15,081 | 14,961 | 14,975 | **18,535** |
| **Ole-CM2** | 15,546 | 15,677 | 15,659 | **19,324** |
| **Ole-CM3** | 15,379 | 15,350 | 15,146 | **19,261** |
| **Ole-CM4** | 15,785 | 15,503 | 15,307 | **19,927** |
| **NT-CM1** | 15,148 | 15,189 | 15,243 | **18,813** |
| **NT-CM2** | 15,807 | 15,656 | 15,852 | **19,384** |
| **NT-CM3** | 16,051 | 15,912 | 15,825 | **19,475** |
| **NT-CM4** | 16,404 | 16,478 | 16,154 | **20,941** |

(**b**) Peptides identified using AMT-DB. Rows represent four biological replicates in oleate-treated (Ole-CM#) and untreated (NT-CM#) conditions, and columns represent technical triplicates (1^st^, 2^nd^, and 3^rd^) for each biological replicate. The 30,933 peptides were quantified in 24 LC-MS/MS datasets.

| **# peptides** | **1^st^** | **2^nd^** | **3^rd^** | **Union** |
| --- | --- | --- | --- | --- |
| **Ole-CM1** | 27,118 | 27,123 | 27,041 | **28,849** |
| **Ole-CM2** | 27,495 | 27,576 | 27,564 | **29,329** |
| **Ole-CM3** | 27,162 | 27,071 | 26,997 | **29,055** |
| **Ole-CM4** | 27,574 | 27,479 | 27,509 | **29,453** |
| **NT-CM1** | 27,186 | 27,212 | 27,228 | **28,959** |
| **NT-CM2** | 27,791 | 27,723 | 27,810 | **29,454** |
| **NT-CM3** | 27,817 | 27,817 | 27,914 | **29,484** |
| **NT-CM4** | 27,404 | 27,565 | 27,571 | **29,364** |
| **Total** |  |  |  | **30,933** |

**Supplementary Table S2**. List of AMTs. For each AMT, its highest MS-GF score in 24 LC-MS/MS datasets, precursor m/z, and charge state (CS) are shown together with UMC intensities in the 24 LC-MS/MS datasets. Four biological replicates in oleate-treated and control conditions were represented by Ole-CM# and control NT-CM#, respectively. Three technical replicates for each biological replicate were denoted by the underscored numbers followed by the index of the biological replicate (e.g., Ole-CM1_1 for 1^st^ technical replicate of Ole-CM1). The ‘16’ in peptide sequences represents oxidation in M, and ‘--’ means that the corresponding peptide was not detected in the sample.

See the attached excel file named “Supplementary Table S2.xlsx”

**Supplementary Table S3**. List of proteins identified from 24 LC-MS/MS datasets. For each protein, the UniProtKB/Swiss-Prot accession and Entrez IDs, symbol, and description are shown together with sequence coverage (%) and the number of identified unique peptides.

See the attached excel file named “Supplementary Table S3.xlsx”

**Supplementary Table S4**. GOBPs represented by the 1,766 secreted protein-encoding genes overlapping with those detected in the previous secretome studies. For each GOBP, the GO term ID and description are shown together with the number of proteins annotated with the GOBP (Count) and the significance of the GOBP being enriched by the overlapping secreted protein-encoding genes (*P* value).

See the attached excel file named “Supplementary Table S4.xlsx”

**Supplementary Table S5**. List of DSPs. For each DSP, the UniProtKB/Swiss-Prot accession and Entrez IDs, symbol, and description are shown together with whether the DSP was up- or down-regulated in oleate-treated condition, compared to controls (D/U), log_2_-fold-changes between oleate-treated and untreated conditions, and the number of unique DEpeptides showing the corresponding up- or down-regulation (Number of unique DEpeptides). The log_2_-fold-changes were calculated as the median log_2_-fold-change of the DEpeptides.

See the attached excel file named “Supplementary Table S5.xlsx”

**Supplementary Table S6**. GOBPs represented by the up-regulated (**a**) and down-regulated secreted proteins (**b**). For each GOBP, the GO term ID and description are shown together with the number of proteins annotated with the GOBP (Count) and the significance of the GOBP being enriched by the up- or down-regulated secreted proteins (*P* value). GOBPs shown in the main figure are highlighted in red (**a**) and green (**b**) backgrounds.

See the attached excel file named “Supplementary Table S6.xlsx”

**Supplementary Table S7**. Associations of the four up-regulated secreted proteins with cancer pathophysiological features. For each protein, the previously reported associations with various types of cancers are shown together with the references.

See the attached excel file named “Supplementary Table S7.xlsx”

**Supplementary Table S8**. Previous cancer secretome studies. In each study, cancer type, the numbers of detected secreted proteins and/or DSPs, compared to controls, and proposed markers are described with the references. Moreover, the table shows which of the six secreted proteins selected in our study was detected or identified as DSPs (up- or down-regulated) in each of the previous cancer secretome studies.

See the attached excel file named “Supplementary Table S8.xlsx”
